# Topical Delivery of Terbinafine HCL Using Nanogels: A New Approach to Superficial Fungal Infection Treatment

**DOI:** 10.3390/gels9110841

**Published:** 2023-10-24

**Authors:** Shams ul Hassan, Ikrima Khalid, Liaqat Hussain, Mohammad T. Imam, Imran Shahid

**Affiliations:** 1 Department of Pharmaceutics, Faculty of Pharmaceutical Sciences, Government College University, Faisalabad 38000, Pakistan; shamsgcuf@gmail.com; 2 Department of Pharmacology, Faculty of Pharmaceutical Sciences, Government College University, Faisalabad 38000, Pakistan; liaqathussaingsk@yahoo.com; 3 Department of Clinical Pharmacy, College of Pharmacy, Prince Sattam Bin Abdulaziz University, Al Kharj 11942, Saudi Arabia; m.imam@psau.edu.sa; 4 Department of Pharmacology and Toxicology, Faculty of Medicine, Umm Al-Qura University, Makkah 21955, Saudi Arabia; iyshahid@uqu.edu.sa

**Keywords:** dermatophytosis, nanogels, terbinafine HCL, acrylic acid, gelatin

## Abstract

This study investigated pH-responsive Terbinafine HCL (TBH)-loaded nanogels as a new approach to treating superficial fungal infections. Acrylic acid (AA) is a synthetic monomer that was crosslinked with a natural polymer (gelatin) using a free radical polymerization technique to fabricate gelatin-g-poly-(acrylic acid) nanogels. Ammonium persulphate (APS) and N, N′-methylene bisacrylamide (MBA) were used as the initiator and crosslinker, respectively. Developed gelatin-g-poly-(acrylic acid) nanogels were evaluated for the swelling study (pH 1.2, 5, 7.4), DEE, particle size, FTIR, thermal stability (TGA, DSC), XRD, SEM, DEE, and in vitro drug release study to obtain optimized nanogels. Optimized nanogels were incorporated into 1% HPMC gel and then evaluated in comparison with Lamisil cream 1% for TBH stratum corneum retention, skin irritation, and in vitro and in vivo antifungal activity studies. Optimized nanogels (AAG 7) demonstrated a 255 nm particle size, 82.37% DEE, pH-dependent swelling, 92.15% of drug release (pH) 7.4 within 12 h, and a larger zone of inhibition compared to Lamisil cream. HPMC-loaded nanogels significantly improved the TBH skin retention percentage, as revealed by an ex vivo skin retention study, indicating the usefulness of nanogels for topical use. In vivo studies conducted on animal models infected with a fungal infection have further confirmed the effectiveness of nanogels compared with the Lamisil cream. Hence, Gelatin-g-poly-(acrylic acid) nanogels carrying poorly soluble TBH can be a promising approach for treating superficial fungal infections.

## 1. Introduction

It is believed that about a billion individuals worldwide suffer from dermatophytosis, or fungal infections of the keratin in skin, hair, and nails, making it one of the most prevalent illnesses of the skin. According to the most prominent international fungal education (LIFE) portal, over 80% of people are susceptible to developing fungal infections [1,2]. Worldwide, dermatophytes are the prevailing causes of fungal infections, and they are most prevalent in hot and moist environments of tropical and subtropical regions such as Pakistan [3,4]. The prevalence and incidence of dermatophytosis fluctuate according to socioeconomic and geographic factors. The economic burden of dermatophytosis can be measured using disability-adjusted life years (DALYs: the sum of the years of potential life lost due to an illness and the years spent living with some disability caused by the disease). Skin diseases caused by fungi are the most common type of skin problem and are accountable for a significant portion of the total DALYs. Compared with various other diseases, incidences of fungal skin infections are the fourth highest among all diseases [5,6]. In addition, dermatophytosis imposes a substantial financial burden on affected communities.

Topical antifungal products are frequently favored to treat epidermal fungal infections over oral medication because of the advantages of self-administration, avoiding first-pass metabolism, better patient compliance, safety, and ease of medication termination. If optimal drug release and penetration are guaranteed, the needed amount for fungicidal action at the skin target area may be more easily attained following topical administration. A higher oral dose is usually required to achieve the same local drug levels, raising the likelihood of unwanted effects. In most cases, when a drug is applied topically, the resulting systemic levels are so low that they are undetected, lowering the drug’s potential toxicity [7]. Nanogels have emerged as the nanoparticle drug delivery system with the most potential among the different dosage forms investigated over the previous twenty years. Nanogels are hydrogel systems made of nanoscale particles synthesized by physical or chemical crosslinked polymer networks that can absorb significant quantities of water or physiological fluids without disturbing the gel network structure. As nanogels are the nanoscale counterparts of hydrogels, they have the characteristics of both hydrogels and nanoparticles. The size of nanogels can vary from 1 to 200 nm. Due to their nanometric dimensions, biocompatibility, biodegradability, tunable size, enormous surface area, stability, high drug encapsulation capacity, viscoelasticity, easy preparation, excellent penetration properties, and stimuli responsiveness, they have gained widespread recognition as a smart drug delivery systems [8,9]. Gelatin is derived from collagen and is an excellent candidate for natural polymer-based nanogels due to its porous structure, solubility, transparency, and biocompatibility. As with many biopolymers, it has limitations, such as weak mechanical characteristics, and requires significant crosslinking (physical or chemical) to be beneficial [10]. Hydrogels with a high water absorbing capacity can be made from AA by crosslinking the monomer in a one- or multi-component system. The carboxylic acid group of AA, also named as propenoic acid, is attached to a vinyl group. The carboxylic acid group, due to its ability to ionize, can assist in augmenting hydrogel ionic strength and pH sensitiveness. Various types of hydrogels can be made by combining AA monomers with other polymers [11]. TBH is an antifungal drug with activity against *Trichophyton mentagrophytes* and *Trichophyton rubrum*, the primary causative agents for superficial fungal infection. TBH outperforms other classes of antifungals in every measurable way (high mycological cure rates, quick treatment times, low recurrence rates, etc.) because it is effective against a far more comprehensive range of fungus species and has a lower minimum inhibitory concentration (MIC) for each species. TBH’s low penetration rate and low aqueous solubility lengthen the treatment duration. Both oral and topical forms of TBH are marketed commercially for treating dermatophytosis. The adverse effects of TBH include liver toxicity, gastrointestinal problems, and cholestatic dysfunction, and they have been linked to oral administration. For drugs administered topically, insufficient absorption causes treatment times to be stretched out, raising healthcare costs and reducing patient adherence [12,13]. 

This study aimed to develop pH-responsive gelatin and AA crosslinked nanogels, physically and chemically characterize them, and investigate them for their DEE, release studies, skin deposition study, and in vitro and in vivo antifungal activity to be suitable to treat Dermatophytosis.

## 2. Results and Discussion

### 2.1. Particle Size Analysis

**Figure 1:**
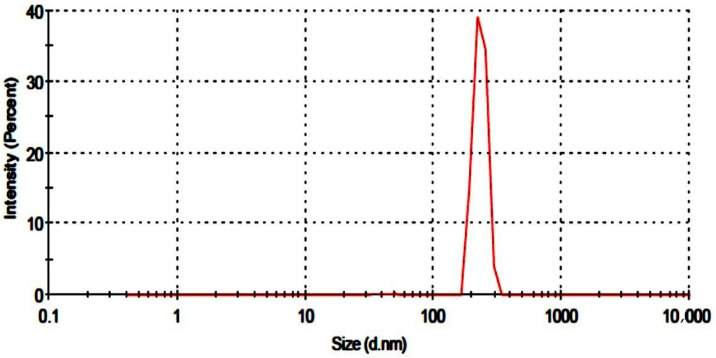
Particle size of developed nanogels.

The particle size of the developed nanogels was assessed using a particle size analyzer (Malvern Zeta Sizer Nano ZS, Malvern, UK). Figure 1 depicts the single zeta sizer curve. The particle diameter was 231.7 nm, and PDI was calculated as 0.245. A low PDI value indicated that fabricated nanogels were homogenous, implying that clusters are less likely to form. Previously gelatin-based nanogels were reported to have a particle size of 255 nm [14]. 

### 2.2. FTIR

**Figure 2:**
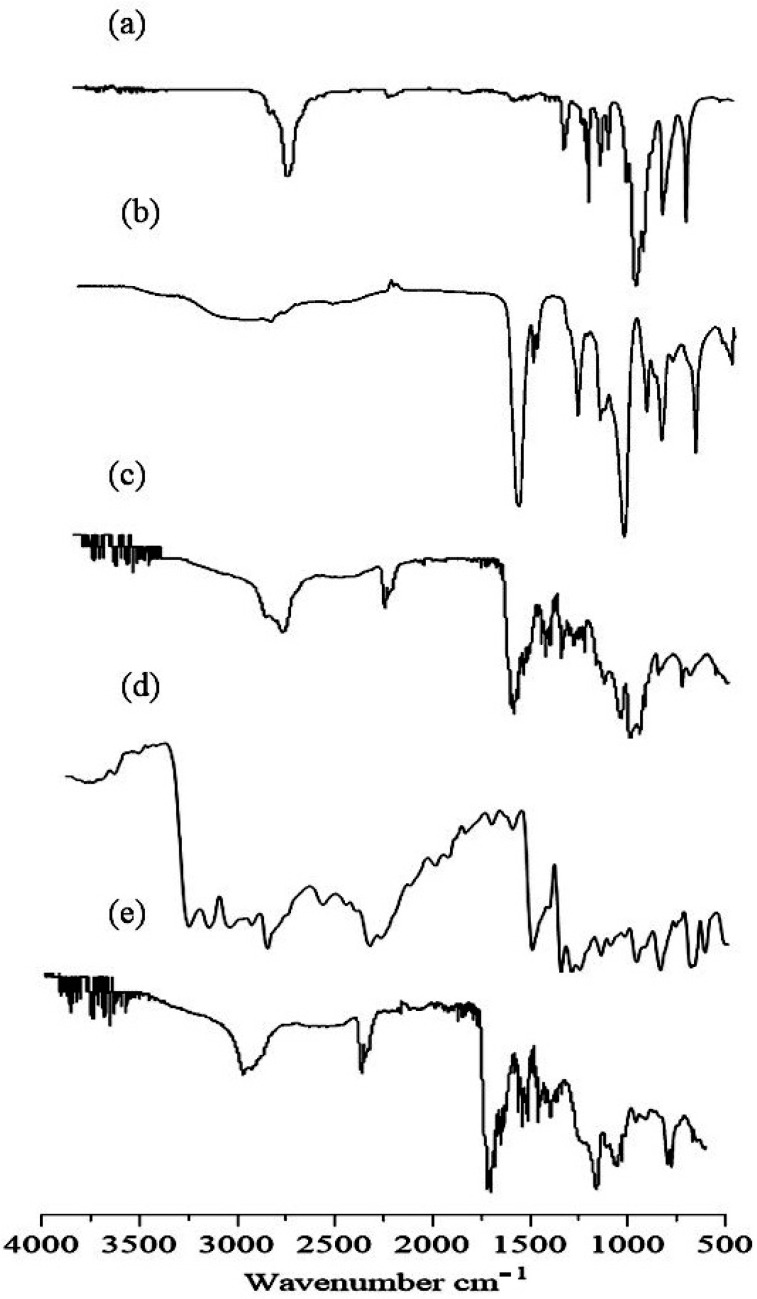
FTIR spectra of (**a**) gelatin, (**b**) AA, (**c**) drug-free nanogel, (**d**) TBH, and (**e**) TBH-loaded nanogel.

IR spectra of gelatin, AA, placebo nanogels, TBH, and TBH-loaded nanogels are presented in Figure 2a–e and were taken in the range of 4000–500 cm^−1^. The spectrum of the gelatin polymer is shown in Figure 2a. Sharp absorption peaks at 2910 cm^−1^ were assigned to C-H stretching, while two characteristic absorption bands at 1665 cm^−1^ and 1541 cm^−1^ appeared for amide I and II, respectively. The IR spectra of AA Figure 2b reported a peak at 2973 cm^−1^ because of the methylene group stretching vibrations. The existence of the COOH group was proved through a peak at 1706 cm^−1^. The peak at 1635 cm^−1^ confirmed the presence of the C=O group, while the peak at 1296 cm^−1^ was assigned to the C-C group. The characteristics absorption band observed at 1173 cm^−1^ verified the stretching vibrations of the COOH group [15,16]. The IR spectra of the developed placebo nanogels in Figure 2c suggest crosslinking between gelatin and AA, as all peaks of gelatin and AA were present with a minor displacement of their frequencies. The peak at 2910 cm^−1^ was due to the C-H stretching vibrations of gelatin, and the peak of AA at 2973 cm^−1^ was overlaid and weakened in the placebo nanogel spectrum. The absorption peaks of amide 1 and II at 1665 cm^−1^ and 1540 cm^−1^ were also shifted to 1675 cm^−1^ and 1547 cm^−1^, respectively. This shifting and weakening of the absorption peaks indicates the chemical interaction between AA and gelatin. Figure 2d shows the IR spectrum of TBH. Some key peaks were observed in the spectrum of TBH at 1408 cm^−1^, 1384 cm^−1^, 2983 cm^−1^, and 3043 cm^−1^, exhibiting the C–N group, CH3 group, aliphatic C–H group, and aromatic C–H groups, respectively [17,18]. In Figure 2e, the IR spectrum of drug-loaded nanogels revealed peaks at 1420 cm^−1^, indicating the existence of the C-N group, while the peak at 1405 cm^−1^ represented the CH_3_ group, and C–H stretching at 2990 cm^−1^ was almost unaffected. It showed that TBH is compatible with the monomer and the polymer. 

### 2.3. TGA

**Figure 3:**
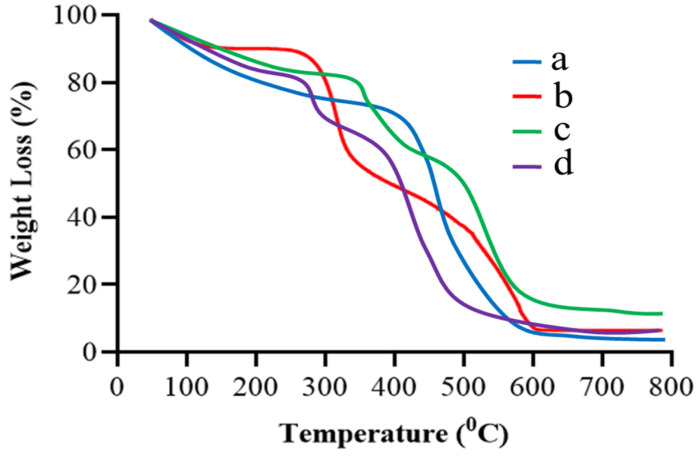
TGA curves of (**a**) gelatin, (**b**) TBH, (**c**) drug-free nanogels, and (**d**) TBH-loaded nanogels.

TGA scan of gelatin in Figure 3a demonstrated three stages of gelatin’s weight reduction. The first stage ranged between 50 and 150 °C and showed about 16% loss in weight. It could be a result of the loss of adsorbed and bound water. In the 150–600 °C temperature range, 70% of the residual polymer decomposed. In the third stage, the leftover polymeric waste was decomposed at 600 to 800 °C [19,20]. In Figure 3b, TBH showed initial degradation in the TGA curve at 195 °C, representing the removal of the drug’s surface assimilated water. Significant degradation occurred between 200 °C and 400 °C, and up to 654 °C, where more than 80% of drug degradation occurred [21]. Figure 3c presents the TGA curve of the naïve nanogels which showed an early mass deprivation of 17% due to water evaporation at 50–350 °C, revealing the higher strength of the formed crosslinked network over the higher temperature value than the individual ingredient. About 22% of weight loss occurred at around 350 °C to 490 °C. The further deterioration of the fabricated nanogels took place in a temperature range of 490 °C to 580 °C. The remaining nanogel weight loss commenced at around 580 °C and continued until complete decay. The TBH-loaded nanogel, depicted in Figure 3d, showed overall good thermal stability and only 17% weight loss until 250 °C, which is due to the strong bonding between the formulation components. The further degradation of the fabricated nanogels took place in the temperature range of 250 to 500 °C. 

### 2.4. DSC

**Figure 4:**
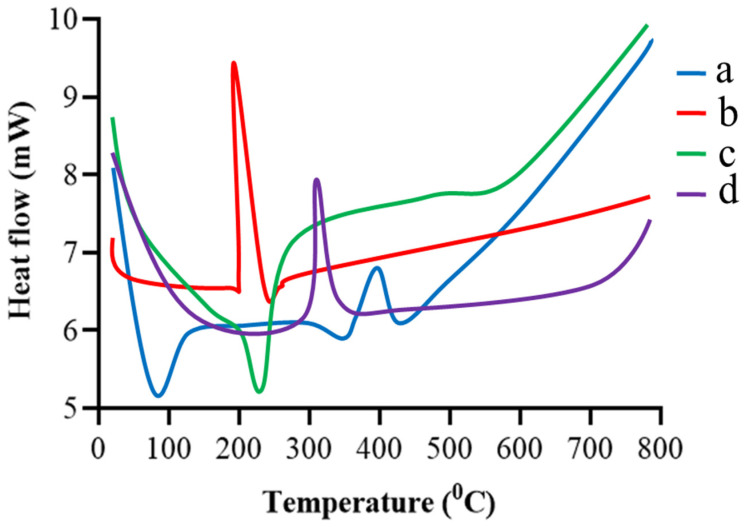
DSC thermogram of (**a**) gelatin, (**b**) TBH, (**c**) drug-free nanogel, and (**d**) TBH-loaded nanogel.

The DSC of gelatin, TBH, and blank and drug-loaded nanogels are presented in Figure 4a–d. Gelatin, presented in Figure 4a, shows two endothermic peaks at 98 °C (transition from a glass to a rubber state) and 341 °C, and one exothermic peak at 399 °C (Brownian motion of the main backbone chain) [22,23]. TBH, in Figure 4b, shows an exothermic peak at 211 °C, corresponding to the drug’s melting point [24,25]. The blank nanogel in Figure 4c displays an endothermic peak at 210 °C, indicating the peak of gelatin and other excipients used in the formulation (the interaction of gelatin with acrylic acid). The TBH-loaded nanogels in Figure 4d show only one exothermic peak at 312 °C; the peak of the TBH is not shown, indicating the amorphous nature and uniform distribution of the drug in the developed nanogels. 

### 2.5. XRD

**Figure 5:**
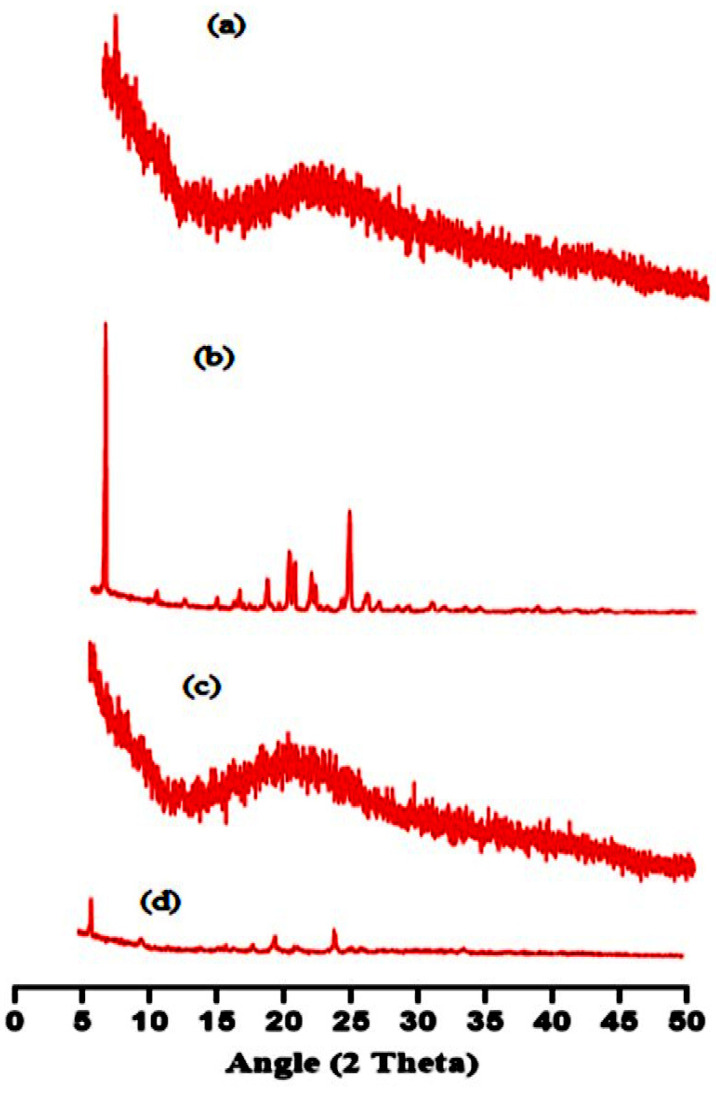
XRD pattern of (**a**) gelatin, (**b**) TBH, (**c**) drug-free nanogel, and (**d**) TBH-loaded nanogel.

The amorphous or crystalline nature of the fundamental ingredients, like pure gelatin, TBH, and both loaded and unloaded nanogels, was determined using an X-ray diffractogram, as shown in Figure 5a–d. The pure gelatin X-ray diffraction patterns in Figure 5a demonstrate that the structure is mainly amorphous with a more noticeable peak at about 20° 2θ [26]. The XRD of TBH in Figure 5b exhibits sharp and prominent peaks at 7°, 18°, 20°, 22°, and 24°, representing its crystalline nature [27,28]. Diffuse peaks rather than sharp ones are seen in the XRD spectra of the unloaded nanogels in Figure 5c. In contrast, in drug-loaded nanogels, Figure 5d shows that the amorphous nature of the carrier sharply reduced the intensity of the drug peaks, proving that the interaction is not the outcome of simple blending, but rather the consequence of chemical bonding between the ingredients and the development of a complex. The resultant fabricated nanogels, due to their amorphous nature, successfully encapsulated TBH inside the formed interconnected network, and the crystalline form of the drug was entirely altered due to crosslinking without showing a drug–excipient interaction. 

### 2.6. Scanning Electron Microscopy (SEM)

**Figure 6:**
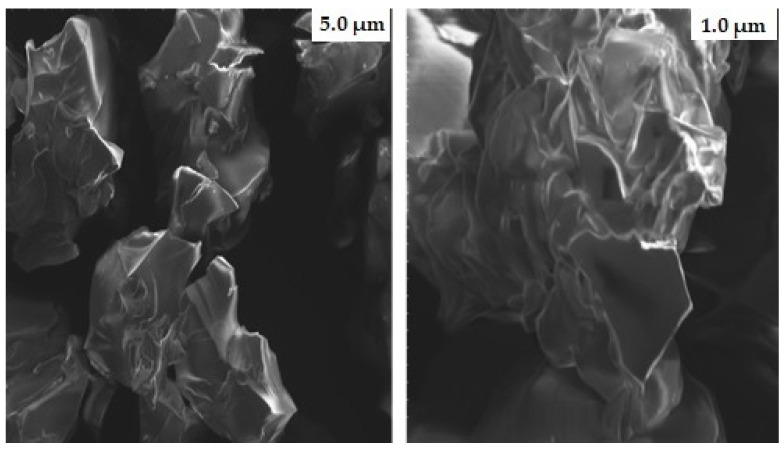
SEM micrographs of gelatin-g-poly-(acrylic acid) nanogels at different magnifications.

The shape of nanogels is crucial as it determines the loading and distribution of drug molecules. SEM photographs were taken to observe the morphological features of fabricated nanogels, as illustrated in Figure 6. The surface of the developed nanogels showed a porous and fluffy appearance. The presence of multiple pores in the nanogels causes them to be more responsive toward the solvent upon interaction. This, in turn, leads to improved drug trapping and the release of the drug from the nanogels. Previously, H. Shaukat et al. found similar outcomes when preparing polymeric nanocomposites for the solubility improvement of rosuvastatin [29].

### 2.7. Swelling Studies

**Figure 7:**
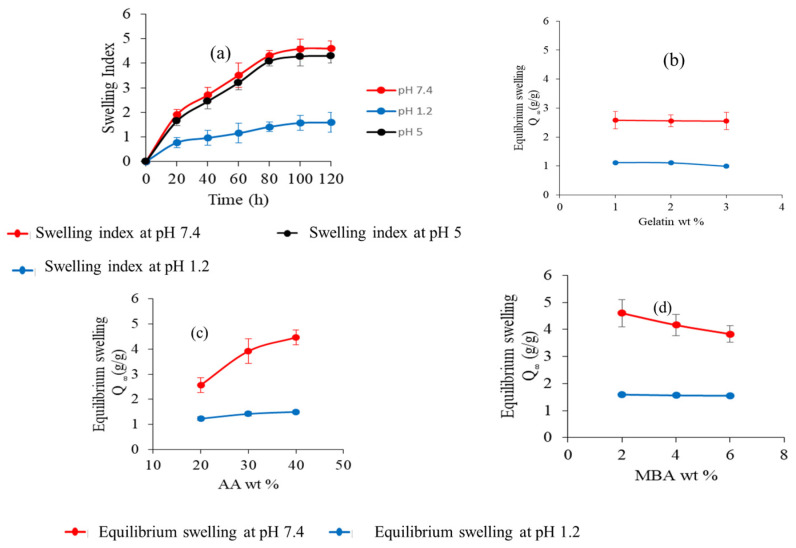
(**a**) Dynamic swelling of nanogels at different pH levels. (**b**) Effect of gelatin wt%. (**c**) Effect of AA wt%. (**d**) Effect of MBA wt% on water absorbency (equilibrium swelling).

The swelling capacity of nanogels with different compositions was investigated in phosphate buffers at pH 1.2, 5, and 7.4. Fabricated nanogels showed an increase in the swelling index when the buffer pH was increased from 1.2 to 5 and 7.4, as depicted in Figure 7a. The pH and pKa value of the polymer are two crucial factors that influence the swelling action of nanogels. The pKa value of AA is about 4.28, and the ionization of its carboxylic acid groups changes with the pH of the buffer. The AA chains collapsed in a phosphate buffer of pH 1.2 because the pH of the medium being higher than the pKa value of AA is necessary for the protonation of their carboxylic group, thus exhibiting no significant swelling. However, in a buffer with pH 5 and 7.4 (more than the pKa value of AA), nanogels start swelling because the carboxylic groups of AA get ionized, resulting in electrostatic repulsion between protonated carboxylic groups in the polymer chain. Ionic osmotic pressure developed inside the polymeric network by counter ions to charged ions also played a vital role in the increase of the swelling index of nanogels. The swelling index of nanogels in buffer with pH 7.4 was slightly higher than in buffer with pH 5 due to the availability of more ionized carboxylic acid groups in the buffer medium. The swelling index of the prepared nanogels decreased as the amount of gelatin increased, as depicted in Figure 7b. Gelatin chains stay protonated below the PI (isoelectric pH) value. As an outcome, the chains contain NH3+ ions, and the cationic repulsion among them may explain their decreased swelling.

However, increasing the gelatin concentration had no definite effect on the swelling of the nanogel [30,31,32]. The swelling index of the nanogels was also affected by the dosage amount of the crosslinking agent. The study results revealed that the swelling of nanogels increased with the increasing amount of AA in both buffer mediums, as shown in Figure 7c. This could be due to increased carboxylic groups of AA for ionization [33,34,35]. The swelling index of nanogels at different concentrations of MBA is shown in Figure 7d. It was observed that as the amount of MBA was increased, the swelling index of nanogels decreased. This decrease in the swelling index can be explained by the reduction of the mesh size of nanogels and the formation of a more compact, highly crosslinked structure. Inside this compact structure, carboxylate groups hide, and the ionization process decreases, which is responsible for nanogels swelling less [36,37]. 

### 2.8. DEE

**Figure 8:**
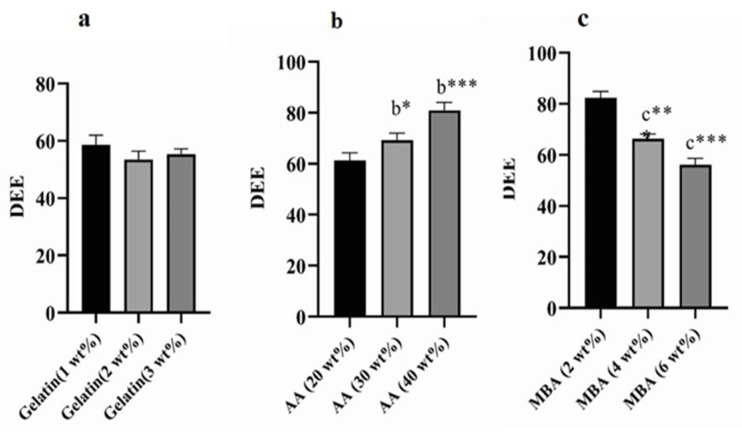
Values are expressed as mean ± SD (n = 3). Results were compared by one-way ANOVA followed by Tukey’s multiple comparison test. (**a**) Denotes significant difference from AAG1. (**b**) Denotes significant difference from AAG4. (**c**) Denotes significant difference from AAG7. * *p* < 0.05, ** *p* < 0.01, *** *p* < 0.001.

The influence of Gelatin, AA, and MBA concentrations on TBH loading in various Gelatin-g-(poly)-Acrylic acid nanogels (AAG1-AAG9) is shown in Figure 8a–c. Swelling behavior and crosslink density are two factors that significantly affect DEE. Nanogels (AAG2 and AAG3) with Gelatin 2 wt% and 3 wt%, respectively, showed an insignificant difference from AAG1 (Gelatin 1 wt%) for DEE with an increasing gelatin content (Figure 8a). However, AAG5 and AAG6, i.e., acrylic acid wt% 30 and 40, respectively, showed a significant increase in DEE compared to AAG4, i.e., acrylic acid 20 wt% (Figure 8b) [38,39]. This enhanced DEE within the polymeric matrix is attributed to the higher swelling of the nanogels. Therefore, DEE and dynamic swelling are directly correlated, i.e., the higher the nanogels’ swelling, the higher their DEE will be. Similarly, a significant decrease in DEE was observed with the increasing concentration of MBA, i.e., 2%, 4%, and 6% (Figure 8c). An increased MBA concentration in nanogels caused an increased crosslinking density between polymeric networks and a decreased water uptake by the nanogel, which resulted in a poor swelling index and lower DEE [36]. 

### 2.9. Drug Release Studies and Kinetics

**Figure 9:**
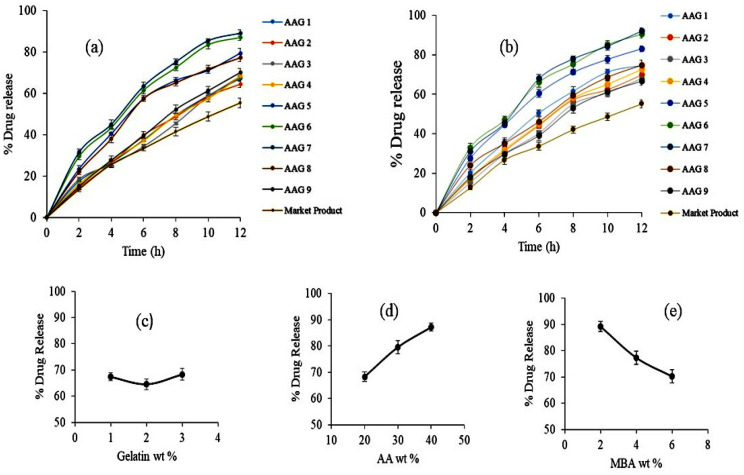
(**a**) Drug release profile from nanogels and the reference product in buffer solution (**a**) pH 5 and (**b**) pH 7.4. (**c**) Effect of gelatin wt% on drug release. (**d**) Effect of AA wt% on drug release. (**e**) Effect of MBA wt% on drug release.

TBH release studies for prepared nanogels (from AAG 1 to AAG 9) and commercial product Lamisil cream 1% were carried out in phosphate buffer solution at pH 5 and 7.4, as shown in Figure 9a,b. The percentage of drug release was higher in the buffer media of pH 7.4 for all developed nanogels. The drug release at pH 7.4 from the developed nanogels ranged from 66.71% to 92.15%. The maximal drug release was exhibited by AAG 7 nanogels which was 92.15%. At pH 7.4, a higher swelling rate of nanogels due to the deprotonation of carboxylic groups of AA to carboxylate ion occurred (pKa of AA = ∼4), resulting in the repulsion and then relaxation of polymer chains; consequently, a greater amount of TBH released from nanogels was observed. The effect of the AA concentration on TBH release from nanogels is shown in Figure 9d. The percentage drug release was higher in the developed nanogels with higher amounts of AA due to the increased drug loading and swelling of the nanogels [30,40,41]. The impact of the amount of gelatin on the percentage drug release from the nanogels (from AAG 1 to AAG 3) is shown in Figure 9c. Although all nanogels had different amounts of gelatin, the percent drug release from the nanogels showed no definite trend with the increasing gelatin concentration. Contrary to AA, the percentage drug release was reduced as the amount of crosslinker (MBA) was enhanced (from AAG 7 to AAG 9) in the nanogels, as shown in Figure 9e. As the feed amount of MBA increased in the nanogel composition, the developed nanogel structure became highly crosslinked; this highly crosslinked compact structure inhibited solvent penetration inside the nanogels, thus reducing both swelling and the percentage of the drug liberated from nanogels. The percentage of the drug release from commercial products compared to our developed nanogels was lesser throughout the study period [42]. 

The pattern of drug release from the loaded nanogels was investigated by applying various kinetic models, i.e., zero-order, first-order, Higuchi, and Korsmeyer–Peppas models, to in vitro drug dissolution data. The results presented in Table 1 indicate that all formulations (F1–F9) displayed a first-order release of drugs with higher R^2^ values when compared with zero-order kinetics at both pH 5 and pH 7.4. Further, a drug release mechanism was assessed by Higuchi and Korsmeyer–Peppas models. From Table 1, it is clear that Korsmeyer–Peppas showed good fitting as all formulations have R^2^ values ranging from 0.9618 to 0.9977 and 0.9713 to 0.928 at pH 5 and pH 7.4, respectively. The Korsmeyer–Peppas model describes the release of drugs from the gels, where ‘‘n’’ is the release exponent that describes the drug release mechanism. When 0.43 ≥ *n*, this corresponds to Fickian diffusion and anomalous release (i.e., non-Fickian diffusion) occurs when 0.43 < *n* < 0.85. For values *n* > 0.85, the release is governed by a super-case-II transport mechanism. The release exponent values for all formulations (F1–F9) ranged from 0.581 to 0.860, concluding that all formulations exhibited anomalous release, i.e., both diffusion and swelling/polymer relaxation.

### 2.10. TBH Skin Penetration and Retention Studies

**Figure 10:**
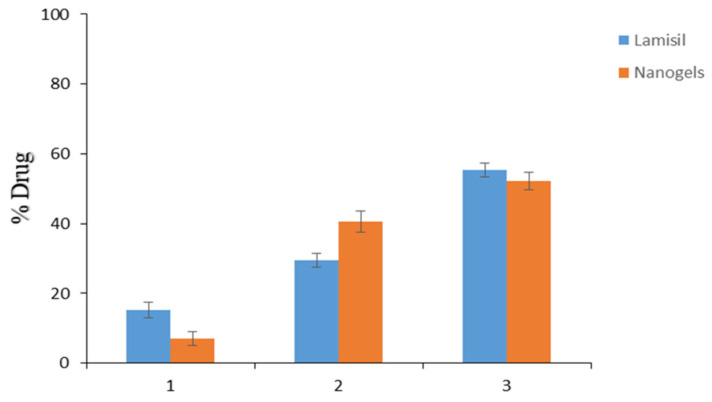
(1) TBH permeated through the skin. (2) TBH deposited in the skin. (3) TBH left on the skin.

Ex vivo skin penetration and retention tests using excised rat skin were performed to estimate if TBH would deposit in the epidermis or permeate transdermally through the skin. TBH-loaded nanogels and Lamisil cream delivered 7.13% and 15.29% of TBH to the receptor compartment, respectively, as shown in Figure 10. Drug–skin permeation from the developed nanogel was less than that of Lamisil cream. It might be due to the lipophilic nature of conventional cream, which penetrates more through the lipophilic structure of corneocytes, resulting in the higher permeation of TBH through the skin [43,44]. After 12 h, 40.57% of the drug from loaded nanogels containing gel was retained in the skin compared to Lamisil cream, which retained only 29.43%. Enhanced drug retention from developed nanogels could be attributed to multiple reasons. One factor for the improved uptake of TBH by skin might result from nanogels’ good spreading and adherence properties at the application site. Smaller size, softness, and deformability properties of nanogels are other vital factors that may contribute to their ability to come into proximity with the SC, enhancing the concentration of drugs that can penetrate the skin. So, it can be said that the carrier (nanogels), its small size, and enormous elastic and deformable particle properties were the main reasons for the excessive TBH retention in the epidermal layers of skin [45,46,47]. By formulating polymeric nanogels, TBH can be delivered at the infection site with lesser systemic access, resulting in minimal complications. Consequently, we can conclude that the topical management of skin infections may greatly benefit from the nanogel drug delivery system.

### 2.11. Antifungal Activity Study

**Figure 11:**
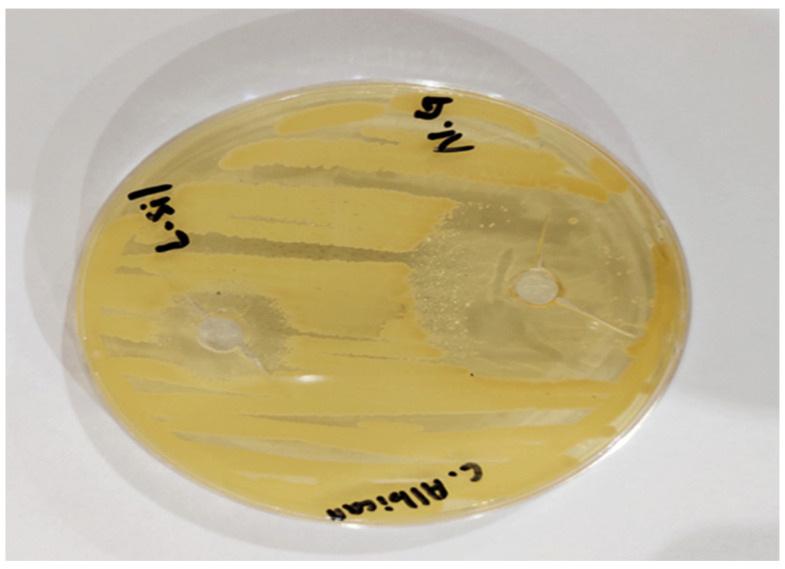
Antifungal activity of nanogels (N.G) in comparison to Lamisil cream (L.sil) against *C. albicans*.

The antifungal activity of synthesized optimized AAG 6 nanogel and the marketed product Lamisil cream was tested against *C. albicans* using the cup–plate method. The zone of inhibition, an indicator of antifungal activity, was measured. A zone of inhibition evaluates an antimicrobial agent’s capacity to stop the growth of microorganisms by observing a clear area around a well containing the antimicrobial substance on the surface of the growth medium. The size of the inhibition zone represents the antimicrobial agent’s strength. TBH-carrying nanogels appeared to be more impactful in killing *C. albicans* compared to Lamisil cream. As shown in Figure 11, the inhibition zone diameter of the drug-loaded nanogel was 32 mm, while the inhibition zone diameter of Lamisil cream was 16 mm. The larger inhibition zone diameter in the case of nanogels might be due to a higher drug release from nanogels and the nanoscale particle size of synthesized nanogels that enhance TBH entry into fungal cells; TBH decreases the concentration of ergosterol by inhibiting fungal enzyme squalene epoxidase and decreased ergosterol synthesis which results in fungal cell death [46,48]. Our antifungal study results are comparable to Y.S.R. Elnaggar et al.’s results, who prepared lecithin-integrated liquid crystalline nanogel loaded with terconazole to treat skin candidiasis [49]. 

### 2.12. Skin Irritancy Studies

Patient acceptability and convenience of topically applied products are limited when the product shows any sign of irritation and erythema on application. Therefore, topically applied dosage forms should be evaluated for their potential for skin irritation and toxicological reactions. The test was performed on rats; Group 2 and Group 3 received formalin and TBH-loaded nanogel-containing gel, respectively. After applying the formulation, the skin was monitored for signs of erythema and edema [50]. Mean erythema and edema scores in the range of 0–4 were recorded, as shown in Table 2, to assess their skin irritation potential. The optimized TBH-loaded nanogel-containing gel scored less than 2 with no severe erythema and edema over 24 h. Products with a score of less than two are considered non-irritant. Therefore, the study’s results disclosed that synthesized nanogels were safe for external application [51].

### 2.13. In Vivo Antifungal Study

**Figure 12:**
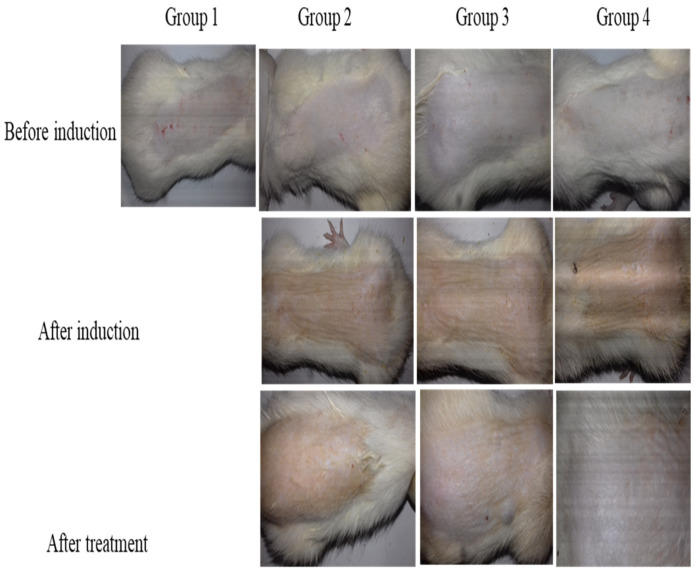
In vivo antifungal activity illustrating skin of rats before fungal infection induction, skin after fungal infection, and skin after treatment.

Fungal skin infection heals when a large amount of drug penetrates inside skin layers and stays on infected skin for longer. An in vivo antifungal cure rate study of drug-loaded nanogels containing bioadhesive HPMC gel compared to commercially available Lamisil cream 1% against a *C. albicans*-induced fungal infection was performed. Before inoculation with *C. albicans*, all four groups of rats presented normal skin without any indication of fungal skin infection, e.g., rashes, redness, swelling, or skin breakout, as shown in Figure 12. Group 1 served as the negative control, while Groups 2, 3, and 4 showed morphological signs of fungal infection, such as yellowish or purple growing skin rashes after inoculation. Group 2 animals received no treatment (positive control) and showed signs of fungal infection throughout the in vivo antifungal study. The animals in Group 3 were treated with commercialized Lamisil cream 1% which relieved skin rashes and cracks, but some yellowish scars did not heal.

In contrast, in animals treated with TBH-loaded nanogels containing bioadhesive gel (Group 4), the manifestation of the fungal infection completely disappeared, and animals recovered normal skin without redness or yellowish marks [52,53,54]. The results revealed that Group 4 rats, which received TBH-loaded nanogel-containing gel, exhibited superior therapeutic outcomes to Group 3 rats which received Lamisil cream 1%. These results could be attributed to a higher penetration of TBH into skin layers when loaded into nanogel-containing gel compared to conventional Lamisil cream.

## 3. Conclusions

Current trends show that fungal diseases are rising, affecting over a billion people annually, either topically or in systemic fungal infections. Nanocarriers like nanogels allow drugs to penetrate the SC following their topical application. The study aimed to investigate the potential of gelatin-g-poly-(acrylic acid) nanogels to serve as carriers for TBH administration through topical routes to enhance its concentration within skin layers. Using a monomer, i.e., AA, and polymer gelatin, pH-responsive nanogels gelatin-g-poly-(acrylic acid) were successfully fabricated using free radical polymerization. Nanogels were fabricated using varying amounts of monomers, polymers, and crosslinkers (MBA). Fabricated nanogels were characterized in terms of their swelling at pH (1.2 and 7.4), particle size, determined interaction between TBH and nanogel components (FTIR), thermal analysis (TGA, DSC), crystalline and amorphous nature of the drug and fabricated nanogels (XRD), and surface morphology (SEM). Following that, nanogels were investigated for their DEE and release studies. In vitro, the antifungal activity on the *C. albicans* strain showed that developed nanogels have a larger zone of inhibition when compared with the Lamisil cream. Ex vivo penetration and retention studies demonstrated that drug-loaded nanogels containing bioadhesive gel could deposit TBH into the skin’s SC layers, which is necessary to treat topical fungal diseases. Skin irritation studies of fabricated nanogel demonstrated that nanogels were non-irritating to the skin. In vivo, antifungal studies in albino rats infected with *C. albicans* revealed that fabricated nanogels better cure the infection than the marketed cream Lamisil.

## 4. Materials and Methods

### 4.1. Materials

AA, MBA, HPMC, and APS were procured from Sigma-Aldrich GmbH, Darmstadt, Germany. Gelatin was purchased from Daejung Chemicals and Metals Co., Ltd., Gyeonggi, Republic of Korea. Sabouraud dextrose agar (SDA) was purchased from Thermofisher Scientific, Waltham, MA, USA. TBH was generously donated by Saffron Pharmaceuticals (Pvt) Ltd., Faisalabad, Pakistan. All other reagents used were analytical grade preparation.

### 4.2. Synthesis of Gelatin-g-Poly-(Acrylic Acid) Nanogels

**Figure 13:**
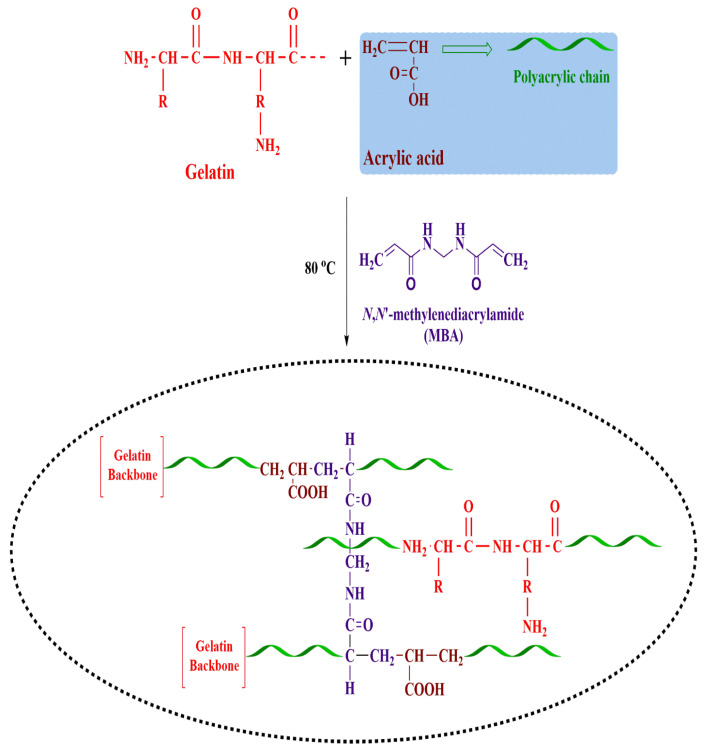
Schematic illustration of fabricated nanogels.

The present work fabricated a series of nanogels (AAG1-AAG9) with different feed compositions using the free radical polymerization technique (Table 3). An aqueous gelatin solution was prepared at room temperature and labeled as solution. (1) A pre-weighted APS (initiator) was dissolved in another beaker in a small amount of water. The initiator solution was added dropwise to accurately weigh AA (monomer), stirred using a magnetic stirrer, and marked as solution. (2) Solution 2 was poured dropwise into solution 1 with continuous stirring at 500 rpm. MBA (crosslinker) solution was prepared in a mixture of water and ethanol at 50 °C using a magnetic stirrer and was added drop by drop to the above reaction mixture while stirring. Nitrogen was continuously supplied to the reaction mixture along with continuous mixing on the hot plate for 45 min to liberate entrapped oxygen. The resultant mixture was homogenized and refluxed at 80 °C to begin the gelation process. The obtained mixture was then washed using a water–ethanol mixture to remove unreacted monomer, sieved, and freeze-dried to obtain nanogels [55,56]. Figure 13 depicts the proposed nanogel structure.

### 4.3. Characterization

#### 4.3.1. Particle Size Analysis

The particle size and particle size distribution of suspension of optimized nanogels in ultrapure-filtered water were evaluated by the Dynamic Laser Scattering method using a particle size analyzer (Malvern Zetasizer Nano ZS, Worcestershire, UK) [43].

#### 4.3.2. FTIR

FTIR analysis was performed for pure drug TBH, gelatin, naïve nanogels, and TBH-loaded nanogels to confirm functional groups of individual ingredients and interactions among the TBH and formulation ingredients. All specimens were spectral scanned over the 4000–500 cm^−1^ scanning range using NICOLET 380 FTIR [57].

#### 4.3.3. Thermal Stability

To determine the thermal stability of components in nanogels, TGA and DSC were conducted on individual components (gelatin and TBH) and developed formulations. A specific amount of sample, ranging from 3 to 5 mg, was placed onto a platinum pan and gradually warmed at 10 °C/min up to 800 °C under the nitrogen environment [46].

#### 4.3.4. XRD

XRD analysis of gelatin, TBH, unloaded, and loaded nanogels was performed to find the crystallinity and amorphicity of samples and results were compared. Diffraction angle 2 (θ) varied from 0 to 50 [50].

#### 4.3.5. SEM

The surface morphology of naïve nanogels was observed using SEM. SEM images were captured at various resolutions after nanogels were affixed on a clear metal stub, sputter coated with gold, and air-dried [58].

#### 4.3.6. Swelling Study

The swelling behavior of all fabricated nanogels was evaluated at different pH conditions (1.2, 5, 7.4) using a dialysis membrane (mol. wt cut-off 14,000). A weighed sample of nanogels was taken into a dialysis bag, submerged separately into buffer solutions of pH 1.2 and 7.4, and nanogels were permitted to swell. The dialysis bag was removed from the buffer solution at a predefined time interval, hung until no liquid oozed out from the bag, and weighed again. The experiment continued until no additional weight gain of nanogels was noticed [59]. The difference in weight represents nanogel swelling. The swelling index of nanogels was calculated using Equation (1).

(1) $$Swelling\;index=\frac{W_{2}}{W_{1}}$$

*W*_1_ denotes the dry weight of the nanogels, and *W*_2_ represents the swollen weight of the nanogel at a particular time (t).

#### 4.3.7. Drug Loading and DEE

TBH loading into the fabricated nanogels was done using the swelling diffusion process. Weighed naïve nanogel was added to the calculated volume of the drug solution prepared by dissolving TBH into the ethanol–water mixture, sonication for 15 min, and then leaving the beaker for 72 h on a magnetic stirrer at 500 rpm. TBH-containing nanogels were air-dried at 25 °C and then subject to lyophilization to remove any solvent [60]. The swelling and extraction method determined the DEE of fabricated nanogels [61]. A weighed amount of TBH-loaded nanogels was transferred to the known amount of hydro-alcoholic mixture and stirred for an hour to get out the entrapped drug from the nanogels. The mixture was centrifuged, and the supernatant layer was collected and filtered through a 0.45 µm membrane filter. In the end, obtained filtrate was evaluated by UV spectrophotometer at *λ*_max_ 283 nm. To find out the DEE, the following equation was used;

(2) $$Drug\;entrapment\;efficiency=\frac{Actual\;drug\;in\;nanogels}{Theoretical\;drug\;in\;nanogels}\times 100$$

#### 4.3.8. Drug Release Studies and Kinetics

In vitro drug release studies for fabricated nanogels compared to the marketed product were carried out using a Franz diffusion cell, consisting of an open-ended cylindrical glass tube, in phosphate buffers (pH 5, 7.4). Previously, phosphate buffer-soaked cellophane membrane was tied to one end of the tube, and the sample of TBH-loaded nanogels was uniformly disseminated on the membrane surface. The whole assembly was left in a beaker containing buffer so that the part of the tube containing nanogels was 1–2 mm below the surface of the buffer. Beaker was placed on a thermostatic hot plate keeping the temperature at 32 ± 0.5 °C and agitation speed at 50 rpm. Suitable samples were withdrawn from the beaker at defined time intervals, and an equivalent quantity of fresh buffer was replaced for each of them. A spectrophotometer was used to determine the drug release at a specific wavelength of 283 nm [62,63]. DSolver Excel add-in program was used to evaluate the release data by zero order, first order, Higuchi, and Korsmeyer–Peppas models.

#### 4.3.9. Preparation of HPMC Gel-Containing TBH-Loaded Nanogels

HPMC gel 1% was prepared for ease of spreadability of solid nanogel particles to rat skin. HPMC, a semisynthetic, inert viscoelastic polymer, was chosen as the gelling agent due to its ability to produce a gel with larger permeability for drug release. Briefly, HPMC was dissolved in distilled water by continuous stirring at 1000 rpm for an hour. Then, accurately weighed TBH-loaded nanogels were mixed thoroughly into the HPMC gel. The pH of the prepared gel was brought up to 5.5 using tri-ethanolamine (TEA) [64].

#### 4.3.10. TBH Skin Penetration and Retention Studies

The ex vivo skin delivery of optimized drug-loaded nanogel-containing gel compared with Lamisil cream 1% onto and across rat skin was carried out using a Franz diffusion cell. Albino rats of either sex, weighing 150–180 g, were utilized for the experiment. Rats were anesthetized and then sacrificed. Rat abdomen skin was excised and washed with pH 7.4 phosphate buffer, cut into appropriate sizes, and hair-free using VEET cream. Subcutaneous tissues and fat were removed without damaging epidermal skin.

The skin in the Franz diffusion cell was put between the donor and receptor compartment so that the skin stratum corneum faced the air and the dermal side of the skin had contact with the receptor compartment fluid. The receptor compartment was filled with phosphate buffer (pH 7.4) and ethanol and continuously mixed during the study period using a magnet at 32 ± 0.5 °C. TBH-loaded nanogel-containing gel and Lamisil cream (equivalent to 3 mg) was added inside the receptor compartment in direct contact with the skin. Samples were periodically removed from the receptor compartment of the Franz diffusion cell and replaced with fresh medium to maintain a constant volume of fluid. Aliquots withdrawn were evaluated for TBH using a UV spectrophotometer preset at *λ*_max_ 283 nm [65].

Ultimately, the skin was removed from the Franz diffusion cell and wiped with a solvent-moistened cotton swab. The cotton swab was extracted and assayed for TBH using a UV spectrophotometer. TBH deposited within skin epidermal layers was determined by chopping the skin. Methanol (i.e., used as a drug extraction solvent) was added to the chopped skin and sonicated to extract TBH into methanol, filtered through a 0.45 µm pore-size membrane filter, and evaluated at 283 nm using a spectrophotometer [66]. The protocol for the study was reviewed and approved by the Ethics Review Committee of Government College University Faisalabad, Faisalabad, Pakistan (GCUF/ERC/268).

#### 4.3.11. Antifungal Activity Studies

The Well method was used to evaluate the antifungal activity of fabricated nanogels against *Candida. Albicans* (*C. albicans*). *C*. *albicans* strain, identified by examining colony morphology, culture characteristics, and VITEK-2 system (Biomerieux, Craponne, France), was provided by the Islamabad Diagnostic Center, Faisalabad, Pakistan. The fungus was maintained on an SDA medium at 4 °C until used in the study. The inoculum was prepared from *C. albicans* and was standardized using 0.5 McFarland standard employing a hemocytometer keeping the final concentration (10^6^ CFU/mL) before the experiment. In total, 1 mL of inoculum was spread onto solidified SDA medium, and two wells were drilled into the media plates. The wells in the plates were filled with Lamisil cream 1% (dissolved into DMSO) and TBH-loaded nanogels (suspended in water) equivalent to 75 µg/mL and incubated plates at 30 °C for 5 days. The zone of inhibition produced was calculated and compared. The experiment was carried out in a clean setting [67,68].

#### 4.3.12. Skin Irritation Studies

The skin irritation tendency of the drug-loaded nanogel-containing gel was evaluated using the Draize test [69]. Albino rats that range in weight from 150 to 180 g were randomly assigned to three groups, each with three rats (*n* = 3). The day before the study, the rats’ dorsal sides were shaved with an electric clipper. Group 1 served as the control group. Group 2 rats received a standard irritant (formalin 1%) solution as the positive control, while drug-loaded nanogels containing gel were topically applied to Group 3 rats. After 24 h, the rats’ used sites were examined for erythema and edema and given a score between 0 and 4 based on the severity of their symptoms [70].

#### 4.3.13. In Vivo Studies

The TBH-loaded nanogel-containing gel’s antifungal activity against *C. albican*’s skin infection was evaluated in albino rats [71]. The weight of the rats was between 150 and 180 g. Rats had easy access to healthy food and water ad libitum and were given a week to adjust to laboratory conditions before the beginning of the study. To establish a *C. albicans* skin infection, rats’ immune systems were suppressed by giving them an intravenous dose of methylprednisolone for three days. To prepare fungal inoculum, *C. albicans* clinical isolates were cultivated on an SDA growth medium and kept in an incubator at 30 °C for five days. A fungal suspension was obtained by adding 3–5 colonies in sterile saline and mixed using a mixer. Using a hemocytometer counter, the fungal concentration was adjusted with sterile saline to 10^7^ CFU/mL. Before inoculation, hairs were removed from the back of animals, the skin was gently scrubbed with ethyl alcohol, and a suspension containing 10^7^ CFU/mL of *C. albicans* in saline was administered intradermally to rats in the middle of exposed skin. After the injection, the swelled area was massaged till the minor edema vanished. A fungal infection on the skin was established after five days. Four rat groups were formed, each with six rats. Group 1 rats served as the negative control group (healthy rats). Group 2 rats received no treatment after contracting a fungal infection and were kept as a positive control. Group 3 and 4 rats were topically applied Lamisil cream 1% and TBH-loaded nanogel-containing gel for 10 days, respectively. All rats in Groups 3 and 4 were treated once per day. Macroscopic examination of fungal infection was employed to assess Lamisil cream 1% treatment efficiency and drug-loaded nanogel-containing gel. The Ethics Review Committee of Government College University Faisalabad, Faisalabad, Pakistan (GCUF/ERC/268), examined and approved all rats experiment in the study.

## Figures and Tables

**Table 1:** Release kinetic modeling.

Formulation Code	pH	Zero Order	First Order	Higuchi Model	Korsmeyer–Peppas Model	
		R^2^	R^2^	R^2^	R^2^	n
F1	5	0.9656	0.9849	0.8862	0.9955	0.808
	7.4	0.8753	0.9954	0.9267	0.9802	0.691
F2	5	0.9599	0.9944	0.8853	0.9917	0.802
	7.4	0.9138	0.9953	0.9117	0.9843	0.731
F3	5	0.9694	0.9556	0.8535	0.9794	0.860
	7.4	0.9436	0.9908	0.8908	0.9848	0.779
F4	5	0.9736	0.9850	0.8803	0.9977	0.825
	7.4	0.9313	0.9946	0.9083	0.9906	0.750
F5	5	0.7687	0.9938	0.9524	0.9732	0.615
	7.4	0.7050	0.9980	0.9693	0.9800	0.581
F6	5	0.7683	0.9868	0.9618	0.9833	0.611
	7.4	0.6756	0.9862	0.9776	0.9852	0.567
F7	5	0.7341	0.9834	0.9668	0.9819	0.594
	7.4	0.7284	0.9802	0.9582	0.9713	0.594
F8	5	0.7910	0.9903	0.9364	0.9618	0.634
	7.4	0.8646	0.9865	0.9451	0.9928	0.671
F9	5	0.9782	0.9849	0.8722	0.9973	0.841
	7.4	0.9277	0.9915	0.9101	0.9898	0.745

**Table 2:** Skin irritancy study outcomes.

Groups		Score ± SD
Group 1 (No application)	Erythema	0.00 ± 0.00
	Edema	0.00 ± 0.00
Group 2 (Formalin)	Erythema	2.66 ± 0.57
	Edema	2.33 ± 0.57
Group 3 (nanogels)	Erythema	0.66 ± 0.57
	Edema	0.33 ± 0.57

Erythema grade set as 0 no erythema, 1 slight, 2 well-defined, 3 moderate, and 4 scar formation. Edema grade set as 0 no edema, 1 slight, 2 well-defined, 3 moderate, and 4 severe.

**Table 3:** Ingredients of Gelatin-g-poly-(acrylic acid) nanogels.

Code	Gelatin (%*w*/*w*)	A.A (%*w*/*w*)	APS (%*w*/*w*)	MBA (%*w*/*w*)
AAG 1	1	20	0.40	2
AAG 2	2	20	0.40	2
AAG 3	3	20	0.40	2
AAG 4	2	20	0.40	2
AAG 5	2	30	0.40	2
AAG 6	2	40	0.40	2
AAG 7	2	40	0.40	2
AAG 8	2	40	0.40	4
AAG 9	2	40	0.40	6

## Data Availability

Data used to support the finding is available on demand from corresponding author on request.

## References

[1] Bongomin F., Gago S., Oladele R.O., Denning D.W. (2017). Global and multi-national prevalence of fungal diseases—Estimate precision. J. Fungi.

[2] Urban K., Chu S., Scheufele C., Giesey R.L., Mehrmal S., Uppal P., Delost G.R. (2021). The global, regional, and national burden of fungal skin diseases in 195 countries and territories: A cross-sectional analysis from the Global Burden of Disease Study 2017. JAAD Int..

[3] Aman S., Haroon T., Hussain I., Bokhari M., Khurshid K. (2001). Tinea unguium in Lahore, Pakistan. Med. Mycol..

[4] Araya S., Tesfaye B., Fente D. (2020). Epidemiology of dermatophyte and non-dermatophyte fungi infection in Ethiopia. Clin. Cosmet. Investig. Dermatol..

[5] Benedict K., Whitham H.K., Jackson B.R. (2022). Economic Burden of Fungal Diseases in the United States. Open Forum Infectious Diseases.

[6] Drgona L., Khachatryan A., Stephens J., Charbonneau C., Kantecki M., Haider S., Barnes R. (2014). Clinical and economic burden of invasive fungal diseases in Europe: Focus on pre-emptive and empirical treatment of Aspergillus and Candida species. Eur. J. Clin. Microbiol. Infect. Dis..

[7] Garg A., Sharma G.S., Goyal A.K., Ghosh G., Si S.C., Rath G. (2020). Recent advances in topical carriers of anti-fungal agents. Heliyon.

[8] Keskin D., Zu G., Forson A.M., Tromp L., Sjollema J., van Rijn P. (2021). Nanogels: A novel approach in antimicrobial delivery systems and antimicrobial coatings. Bioact. Mater..

[9] Shah S., Rangaraj N., Laxmikeshav K., Sampathi S. (2020). Nanogels as drug carriers–Introduction, chemical aspects, release mechanisms and potential applications. Int. J. Pharm..

[10] Chen Y. (2019). Hydrogels Based on Natural Polymers.

[11] Sennakesavan G., Mostakhdemin M., Dkhar L., Seyfoddin A., Fatihhi S. (2020). Acrylic acid/acrylamide based hydrogels and its properties-A review. Polym. Degrad. Stab..

[12] Darkes M.J., Scott L.J., Goa K.L. (2003). Terbinafine. Am. J. Clin. Dermatol..

[13] Karri V., Raman S.K., Kuppusamy G., Mulukutla S., Ramaswamy S., Malayandi R. (2015). Terbinafine hydrochloride loaded nanoemulsion based gel for topical application. J. Pharm. Investig..

[14] Koul V., Mohamed R., Kuckling D., Adler H.-J.P., Choudhary V. (2011). Interpenetrating polymer network (IPN) nanogels based on gelatin and poly (acrylic acid) by inverse miniemulsion technique: Synthesis and characterization. Colloids Surf. B Biointerfaces.

[15] Ajaz N., Khan I.U., Khalid I., Khan R.U., Khan H.A., Asghar S., Khalid S.H., Shahzad Y., Yousaf A.M., Hussain T. (2020). In vitro and toxicological assessment of dexamethasone sodium phosphate loaded pH sensitive Pectin-g-poly (AA)/PVP semi interpenetrating network. Mater. Today Commun..

[16] Malik N.S., Ahmad M., Minhas M.U. (2017). Cross-linked β-cyclodextrin and carboxymethyl cellulose hydrogels for controlled drug delivery of acyclovir. PLoS ONE.

[17] Kuminek G., Rauber G.S., Riekes M.K., de Campos C.E.M., Monti G.A., Bortoluzzi A.J., Cuffini S.L., Cardoso S.G. (2013). Single crystal structure, solid state characterization and dissolution rate of terbinafine hydrochloride. J. Pharm. Biomed. Anal..

[18] Patel M.M., Vora Z.M. (2016). Formulation development and optimization of transungual drug delivery system of terbinafine hydrochloride for the treatment of onychomycosis. Drug Deliv. Transl. Res..

[19] Sadeghi M., Heidari B. (2011). Crosslinked graft copolymer of methacrylic acid and gelatin as a novel hydrogel with pH-responsiveness properties. Materials.

[20] Zheng J.P., Li P., Ma Y.L., Yao K.D. (2002). Gelatin/montmorillonite hybrid nanocomposite. I. Preparation and properties. J. Appl. Polym. Sci..

[21] Sun B., Zhang M., Zhou N., Chu X., Yuan P., Chi C., Wu F., Shen J. (2018). Study on montmorillonite–chlorhexidine acetate–terbinafine hydrochloride intercalation composites as drug release systems. RSC Adv..

[22] Mendieta-Taboada O., Sobral P.J.d.A., Carvalho R.A., Habitante A.M.B. (2008). Thermomechanical properties of biodegradable films based on blends of gelatin and poly (vinyl alcohol). Food Hydrocoll..

[23] Rafique N., Ahmad M., Minhas M.U., Badshah S.F., Malik N.S., Khan K.U. (2022). Designing gelatin-based swellable hydrogels system for controlled delivery of salbutamol sulphate: Characterization and toxicity evaluation. Polym. Bull..

[24] Hossain A.M.A., Sil B.C., Iliopoulos F., Lever R., Hadgraft J., Lane M.E. (2019). Preparation, characterisation, and topical delivery of terbinafine. Pharmaceutics.

[25] Šnejdrová E., Martiška J., Loskot J., Paraskevopoulos G., Kováčik A., Regdon Jr G., Budai-Szűcs M., Palát K., Konečná K. (2021). PLGA based film forming systems for superficial fungal infections treatment. Eur. J. Pharm. Sci..

[26] Radev L., Fernandes M., Salvado I., Kovacheva D. (2009). Organic/Inorganic bioactive materials Part III: In vitro bioactivity of gelatin/silicocarnotite hybrids. Open Chem..

[27] Hajare A., Dol H., Patil K. (2021). Design and development of terbinafine hydrochloride ethosomal gel for enhancement of transdermal delivery: In vitro, in vivo, molecular docking, and stability study. J. Drug Deliv. Sci. Technol..

[28] Kumar P., Mohan C., Shankar M.K.U., Gulati M. (2011). Physiochemical characterization and release rate studies of soliddispersions of ketoconazole with pluronic f127 and pvp k-30. Iran. J. Pharm. Res. IJPR.

[29] Shoukat H., Pervaiz F., Khan M., Rehman S., Akram F., Abid U., Noreen S., Nadeem M., Qaiser R., Ahmad R. (2022). Development of β-cyclodextrin/polyvinypyrrolidone-co-poly (2-acrylamide-2-methylpropane sulphonic acid) hybrid nanogels as nano-drug delivery carriers to enhance the solubility of Rosuvastatin: An in vitro and in vivo evaluation. PLoS ONE.

[30] Bukhari S.M.H., Khan S., Rehanullah M., Ranjha N.M. (2015). Synthesis and characterization of chemically cross-linked acrylic acid/gelatin hydrogels: Effect of pH and composition on swelling and drug release. Int. J. Polym. Sci..

[31] Majeed A., Pervaiz F., Shoukat H., Shabbir K., Noreen S., Anwar M. (2020). Fabrication and evaluation of pH sensitive chemically cross-linked interpenetrating network [Gelatin/Polyvinylpyrrolidone-co-poly (acrylic acid)] for targeted release of 5-fluorouracil. Polym. Bull..

[32] Oh J., Kim B. (2020). Mucoadhesive and pH-responsive behavior of gelatin containing hydrogels for protein drug delivery applications. Korea-Aust. Rheol. J..

[33] Abd El-Rehim H.A., Hegazy E.-S.A., Hamed A.A., Swilem A.E. (2013). Controlling the size and swellability of stimuli-responsive polyvinylpyrrolidone–poly (acrylic acid) nanogels synthesized by gamma radiation-induced template polymerization. Eur. Polym. J..

[34] Chen Y., Zheng X., Qian H., Mao Z., Ding D., Jiang X. (2010). Hollow core–porous shell structure poly (acrylic acid) nanogels with a superhigh capacity of drug loading. ACS Appl. Mater. Interfaces.

[35] Mackiewicz M., Stojek Z., Karbarz M. (2019). Synthesis of cross-linked poly (acrylic acid) nanogels in an aqueous environment using precipitation polymerization: Unusually high volume change. R. Soc. Open Sci..

[36] Ashri A., Lazim A. (2014). A study on the effect of the concentration of N,N-methylenebisacrylamide and acrylic acid toward the properties of *Dioscorea hispida*-starch-based hydrogel. AIP Conf. Proc..

[37] Bhadani R., Mitra U.K. (2016). Synthesis and studies on water swelling behaviour of polyacrylamide hydrogels. Macromol. Symp..

[38] Ali L., Ahmad M., Aamir M.N., Minhas M.U., Shah H.H., Shah M.A. (2020). Cross-linked pH-sensitive pectin and acrylic acid based hydrogels for controlled delivery of metformin. Pak. J. Pharm. Sci..

[39] Mackiewicz M., Dagdelen S., Marcisz K., Waleka-Bargiel E., Stojek Z., Karbarz M. (2021). Redox-degradable microgel based on poly (acrylic acid) as drug-carrier with very high drug-loading capacity and decreased toxicity against healthy cells. Polym. Degrad. Stab..

[40] Jaiswal M., Naz F., Dinda A.K., Koul V. (2013). In vitro and in vivo efficacy of doxorubicin loaded biodegradable semi-interpenetrating hydrogel implants of poly (acrylic acid)/gelatin for post surgical tumor treatment. Biomed. Mater..

[41] Raafat A.I. (2010). Gelatin based pH-sensitive hydrogels for colon-specific oral drug delivery: Synthesis, characterization, and in vitro release study. J. Appl. Polym. Sci..

[42] Kabiri K., Omidian H., Hashemi S., Zohuriaan-Mehr M. (2003). Synthesis of fast-swelling superabsorbent hydrogels: Effect of crosslinker type and concentration on porosity and absorption rate. Eur. Polym. J..

[43] Avasatthi V., Pawar H., Dora C.P., Bansod P., Gill M.S., Suresh S. (2016). A novel nanogel formulation of methotrexate for topical treatment of psoriasis: Optimization, in vitro and in vivo evaluation. Pharm. Dev. Technol..

[44] Mohammed N., Rejinold N.S., Mangalathillam S., Biswas R., Nair S.V., Jayakumar R. (2013). Fluconazole loaded chitin nanogels as a topical ocular drug delivery agent for corneal fungal infections. J. Biomed. Nanotechnol..

[45] Datt N., Poonuru R.R., Yadav P.K. (2022). Development and Characterization of Griseofulvin Loaded nanostructured lipid carrier gel for Treating Dermatophytosis. Food Hydrocoll. Health.

[46] Farooq U., Rasul A., Sher M., Qadir M.I., Nazir I., Mehmood Y., Riaz H., Shah P.A., Jamil Q.A., Khan B.A. (2020). Development, characterization and evaluation of anti-fungal activity of miconazole based nanogel prepared from biodegradable polymer. Pak. J. Pharm. Sci..

[47] Giulbudagian M., Yealland G., Hönzke S., Edlich A., Geisendörfer B., Kleuser B., Hedtrich S., Calderón M. (2018). Breaking the barrier-potent anti-inflammatory activity following efficient topical delivery of etanercept using thermoresponsive nanogels. Theranostics.

[48] de Carvalho S.Y.B., Almeida R.R., Pinto N.A.R., de Mayrinck C., Vieira S.S., Haddad J.F., Leitao A.A., Guimaraes L.G.d.L. (2021). Encapsulation of essential oils using cinnamic acid grafted chitosan nanogel: Preparation, characterization and antifungal activity. Int. J. Biol. Macromol..

[49] Elnaggar Y.S., Talaat S.M., Bahey-El-Din M., Abdallah O.Y. (2016). Novel lecithin-integrated liquid crystalline nanogels for enhanced cutaneous targeting of terconazole: Development, in vitro and in vivo studies. Int. J. Nanomed..

[50] Divya G., Panonnummal R., Gupta S., Jayakumar R., Sabitha M. (2016). Acitretin and aloe-emodin loaded chitin nanogel for the treatment of psoriasis. Eur. J. Pharm. Biopharm..

[51] Elkomy M.H., Elmenshawe S.F., Eid H.M., Ali A.M. (2016). Topical ketoprofen nanogel: Artificial neural network optimization, clustered bootstrap validation, and in vivo activity evaluation based on longitudinal dose response modeling. Drug Deliv..

[52] Beyki M., Zhaveh S., Khalili S.T., Rahmani-Cherati T., Abollahi A., Bayat M., Tabatabaei M., Mohsenifar A. (2014). Encapsulation of Mentha piperita essential oils in chitosan–cinnamic acid nanogel with enhanced antimicrobial activity against *Aspergillus flavus*. Ind. Crops Prod..

[53] Mori N.M., Patel P., Sheth N.R., Rathod L.V., Ashara K.C. (2017). Fabrication and characterization of film-forming voriconazole transdermal spray for the treatment of fungal infection. Bull. Fac. Pharm. Cairo Univ..

[54] Somagoni J., Boakye C.H., Godugu C., Patel A.R., Mendonca Faria H.A., Zucolotto V., Singh M. (2014). Nanomiemgel-a novel drug delivery system for topical application-in vitro and in vivo evaluation. PLoS ONE.

[55] Khalid Q., Ahmad M., Minhas M.U., Batool F., Malik N.S., Rehman M. (2021). Novel β-cyclodextrin nanosponges by chain growth condensation for solubility enhancement of dexibuprofen: Characterization and acute oral toxicity studies. J. Drug Deliv. Sci. Technol..

[56] Sarfraz R.M., Khan M.U., Mahmood A., Akram M.R., Minhas M.U., Qaisar M.N., Ali M.R., Ahmad H., Zaman M. (2020). Synthesis of co-polymeric network of carbopol-g-methacrylic acid nanogels drug carrier system for gastro-protective delivery of ketoprofen and its evaluation. Polym.-Plast. Technol. Mater..

[57] El-Feky G.S., El-Banna S.T., El-Bahy G., Abdelrazek E., Kamal M. (2017). Alginate coated chitosan nanogel for the controlled topical delivery of Silver sulfadiazine. Carbohydr. Polym..

[58] Sahu P., Kashaw S.K., Sau S., Kushwah V., Jain S., Agrawal R.K., Iyer A.K. (2019). pH responsive 5-fluorouracil loaded biocompatible nanogels for topical chemotherapy of aggressive melanoma. Colloids Surf. B Biointerfaces.

[59] Ahmad A., Ahmad M., Minhas M.U., Sarfraz M., Sohail M., Khan K.U., Tanveer S., Ijaz S. (2022). Synthesis and Evaluation of Finasteride-Loaded HPMC-Based Nanogels for Transdermal Delivery: A Versatile Nanoscopic Platform. BioMed Res. Int..

[60] Rao K.M., Mallikarjuna B., Rao K.K., Siraj S., Rao K.C., Subha M. (2013). Novel thermo/pH sensitive nanogels composed from poly (N-vinylcaprolactam) for controlled release of an anticancer drug. Colloids Surf. B Biointerfaces.

[61] Sahu P., Kashaw S.K., Kashaw V., Shabaaz J., Dahiya R. (2021). Synthesis and ex vivo evaluation of PLGA chitosan surface modulated double walled transdermal Pluronic nanogel for the controlled delivery of Temozolomide. Int. J. Biol. Macromol..

[62] Rajput R.L., Narkhede J.S., Mujumdar A., Naik J.B. (2020). Synthesis and evaluation of luliconazole loaded biodegradable nanogels prepared by pH-responsive Poly (acrylic acid) grafted Sodium Carboxymethyl Cellulose using amine based cross linker for topical targeting: In vitro and Ex vivo assessment. Polym.-Plast. Technol. Mater..

[63] Arafa M.G., Ayoub B.M. (2017). DOE optimization of nano-based carrier of pregabalin as hydrogel: New therapeutic & chemometric approaches for controlled drug delivery systems. Sci. Rep..

[64] Cai X.J., Mesquida P., Jones S.A. (2016). Investigating the ability of nanoparticle-loaded hydroxypropyl methylcellulose and xanthan gum gels to enhance drug penetration into the skin. Int. J. Pharm..

[65] Chen Y.-C., Liu D.-Z., Liu J.-J., Chang T.-W., Ho H.-O., Sheu M.-T. (2012). Development of terbinafine solid lipid nanoparticles as a topical delivery system. Int. J. Nanomed..

[66] Vaghasiya H., Kumar A., Sawant K. (2013). Development of solid lipid nanoparticles based controlled release system for topical delivery of terbinafine hydrochloride. Eur. J. Pharm. Sci..

[67] Abbaszadeh S., Rashidipour M., Khosravi P., Shahryarhesami S., Ashrafi B., Kaviani M., Sarabi M.M. (2020). Biocompatibility, cytotoxicity, antimicrobial and epigenetic effects of novel chitosan-based quercetin nanohydrogel in human cancer cells. Int. J. Nanomed..

[68] Wavikar P., Vavia P. (2013). Nanolipidgel for enhanced skin deposition and improved antifungal activity. AAPS Pharmscitech.

[69] Ghose A., Nabi B., Rehman S., Md S., Alhakamy N.A., Ahmad O.A., Baboota S., Ali J. (2020). Development and Evaluation of Polymeric Nanosponge Hydrogel for Terbinafine Hydrochloride: Statistical Optimization, In Vitro and In Vivo Studies. Polymers.

[70] Morteza-Semnani K., Saeedi M., Akbari J., Hedayati S., Hashemi S.M.H., Rahimnia S.M., Babaei A., Ghazanfari M., Haghani I., Hedayati M.T. (2022). Green Formulation, characterization, antifungal and biological safety evaluation of Terbinafine HCl niosomes and niosomal gels manufactured by eco-friendly green method. J. Biomater. Sci. Polym. Ed..

[71] Rarokar N.R., Menghani S.S., Kerzare D.R., Khedekar P.B., Bharne A.P., Alamri A.S., Alsanie W.F., Alhomrani M., Sreeharsha N., Asdaq S.M.B. (2022). Preparation of Terbinafin-Encapsulated Solid Lipid Nanoparticles Containing Antifungal Carbopol^®^ Hydrogel with Improved Efficacy: In Vitro, Ex Vivo and In Vivo Study. Pharmaceutics.

